# Drug retention and safety of TNF inhibitors in elderly patients with rheumatoid arthritis

**DOI:** 10.1186/s12891-016-1185-6

**Published:** 2016-08-09

**Authors:** Soo-Kyung Cho, Yoon-Kyoung Sung, Dam Kim, Soyoung Won, Chan-Bum Choi, Tae-Hwan Kim, Jae-Bum Jun, Dae-Hyun Yoo, Sang-Cheol Bae

**Affiliations:** 1 Department of Rheumatology, Hanyang University Hospital for Rheumatic Diseases, Seoul, 133-792 South Korea; 2 Clinical Research Center for Rheumatoid Arthritis, Seoul, South Korea

**Keywords:** Rheumatoid arthritis, TNF inhibitor, Elderly, Safety, Drug retention rate

## Abstract

**Background:**

The concerns about the development of adverse events (AEs) in elderly RA patients as a result of age-related changes in drug metabolism and the presence of comorbid illnesses are emphasizing due to increasing prevalence of rheumatoid arthritis (RA) in old age. However, they tend to be inadequately represented in RA clinical trials because of the exclusion criteria that are commonly applied. The tolerability and safety of TNF inhibitors in elderly patients have not been also evaluated in clinical practice. This study aimed to evaluate the retention rate and safety of TNF inhibitors (TNFI) in elderly RA patients.

**Methods:**

Total 429 RA patients (838 person-years [PYs]) treated with TNFI from a retrospective biologic DMARDs registry. Patients were divided into an elderly (age ≥60 years) and a younger group (<60 years). The drug retention rates of both groups were compared using Kaplan-Meier curves. Potential predictors of TNFI discontinuation in the elderly were examined using Cox regression analysis. The incidence rate (IR) of serious adverse events (SAEs) in the elderly group was compared to that of the young group.

**Results:**

Of the patients, 24.9 % (*n* = 107, 212 PYs) were in the elderly group. Regarding the retention rates of TNFI in 3 years, there was no significant difference between the elderly and younger group (*p* = 0.33). The major cause of discontinuation in elderly patients was AE (34.3 %), whereas that was drug ineffectiveness (41.7 %) in younger patients. Age (HR 1.09, CI 1.02-1.16) was a predictor of discontinuation, while the presence of comorbidity (HR 0.37, CI 0.15-0.91) had a protective effect against drug discontinuation in the elderly. The IR of SAEs in the elderly (6.13/100 PYs) was higher than in the younger group (5.11/100 PYs).

**Conclusions:**

The retention rate of TNFI in the elderly was comparable with that in younger patients. The major cause of discontinuation in the elderly patients was AEs, while it was drug ineffectiveness in younger patients. The IR of SAEs in the elderly was higher than in the younger patients.

**Electronic supplementary material:**

The online version of this article (doi:10.1186/s12891-016-1185-6) contains supplementary material, which is available to authorized users.

## Background

Rheumatoid arthritis (RA) is a chronic inflammatory disease that causes joint destruction and disability. The incidence of RA increases with age, peaking between the fourth and sixth decades [1]. Elderly patients diagnosed after the age of 60 comprise 15–33 % of RA patients [2, 3]. A Finnish observational study suggested that the mean age at diagnosis had increased from 50.2 years old to 57.8 years old over 15 years [4]. Along with increasing life expectancy in the industrialized world, these observations suggest that the number of elderly RA patients will increase [5, 6].

However, elderly patients may be less likely to receive tumor necrosis factor (TNF) inhibitors due to their greater likelihood of comorbid conditions than younger patients, and there are often contraindications to treatment with biologic agents [7, 8]. In addition, there have been concerns that elderly patients have a higher risk of adverse events (AEs) including infection [9]. These factors complicate disease treatment and necessitate careful patient management. Although the treatment goals for elderly RA patients are not different from those of younger individuals, the potential risk of developing drug-related AEs in elderly patients is increasing [7].

Previous clinical trials suggest that disease-modifying anti-rheumatic drugs (DMARDs) and TNF inhibitors are efficacious and well tolerated in elderly patients [10, 11]. However, elderly patients tend to be inadequately represented in RA clinical trials due to the exclusion criteria that are commonly applied [12]. Therefore, patients with RA in clinical practice might differ from patients selected for inclusion in clinical trials. To complement evidence obtained from clinical trials, a long-term observational study that evaluates effectiveness and safety in clinical practice is needed.

This study aimed to examine the retention and safety of TNF inhibitors in elderly patients with RA compared to a younger group of patients with RA in clinical practice.

## Methods

### Participants

#### Data source

A retrospective registry of Korean patients with RA (REtrospective study for Safety and Effectiveness of Anti-RA treatment with biologiCs, RESEARCh) was established to evaluate the safety and effectiveness of biologic DMARDs [13]. Patients who meet the 1987 American College of Rheumatology criteria for RA who had ever been given biologic DMARDs from December 2000 to June 2011 were identified from the medical records of Hanyang University Hospital for Rheumatic Diseases and enrolled in the RESEARCh database. Comprehensive chart reviews for all patients were undertaken by well-trained health professionals from November 2009 to August 2011. Demography, disease activity, comorbidities: cardiovascular disease, pulmonary disease, previous history of pulmonary tuberculosis, gastrointestinal disease, hepatobiliary disease, diabetes mellitus, malignancy, hypertension, thyroid disease, and renal disease, treatments, and laboratory data at the first dose of biologic DMARDs were recorded. Comorbidities were assessed at the time of starting biologic DMARDs and were classified by organ system.

#### Patients and follow-up

A total of 429 RA patients (838 person-years [PYs]) treated with TNF inhibitors were included in this study. Patients were divided into two groups: elderly (age ≥60 years) and younger (age <60 years). The mean observational period was 23.4 ± 23.9 months, with 23.8 ± 25.8 months in the elderly group and 23.3 ± 23.2 months in the younger group.

### Drug retention and discontinuation

The observation period for this study started at the first dose of TNF inhibitors. Observation of each patient was stopped either at discontinuation of TNF inhibitors, switching to other biologic DMARDs, death, loss to follow-up, or enrollment in clinical trials, whichever came first. Treatment discontinuation was defined as stopping TNF inhibitor administration for more than 90 days. Reasons for discontinuing TNF inhibitors were retrieved from medical records and classified into adverse events (AEs), ineffectiveness, good effectiveness, patient need, economic reasons, or miscellaneous.

### Serious adverse events (SAEs)

SAEs, including serious infection (SI), were defined based on a report by the International Conference on Harmonization [14]. Bacterial infections that required intravenous administration of antibiotics, as well as opportunistic infections, were also regarded as SAEs. SAEs were classified using the System Organ Class (SOC) of the medical dictionary for regulatory activities (MedDRA version 11.1). SAEs were attributed to TNF inhibitors when they developed during treatment with these biologics, and no risk window was applied.

### Statistical analysis

A comparison of demographic and clinical features between elderly and younger groups was performed using the chi-square test for categorical variables and the Student t-test for continuous variables. The drug retention rate of the elderly group was estimated using Kaplan-Meier analysis, and it was compared with that of the younger group using a log-rank test. Potential predictors of TNF inhibitor discontinuation were also examined using Cox regression analyses. The incidence rate (IR) of SAEs was evaluated in each group and the incidence rate ratio (IRR) of the elderly group with 95 % confidence intervals (CI) was compared to that of the younger group.

These statistical analyses were conducted using SAS 9.2 (SAS Institute, Cary NC). All p values were two-tailed, and *p* < 0.05 was considered statistically significant.

## Results

### Baseline characteristics of elderly RA patients who started TNF inhibitors

Of 429 RA patients treated with TNF inhibitors, 24.9 % (*n* = 107, 212 PYs) were included in the elderly group and 322 (626 PYs) were included in the younger group. The elderly group had more males than the young group (22.4 % vs. 11.2 %, *p* = 0.01). The elderly group tended to use methotrexate (MTX) at a lower dosage than the younger group (13.6 ± 3.5 mg/week vs. 14.2 ± 3.6 mg/week, *p* = 0.20) and glucocorticoids at a higher dosage (prednisolone (PSL)-equivalent dose, 6.3 ± 4.4 mg/day vs. 6.0 ± 4.1 mg/day, *p* = 0.66), but there was no significant difference. The mean number of previous non-biologic DMARDs was higher as 4.4 ± 1.7 in the elderly group than in the younger group (3.9 ± 1.5, *p* = 0.01).

The prevalence of comorbidities was significantly different between the two groups. The elderly group had more comorbid conditions such as cardiovascular disease (7.5 % for the elderly group vs. 1.2 % for the younger group), pulmonary disease (15.9 % for the elderly group vs. 1.9 % for the younger group), including interstitial lung disease, asthma, and chronic obstructive pulmonary disease. Diabetes mellitus (18.7 % for the elderly group vs. 6.5 % for the younger group), and hypertension (46.7 % for the elderly group vs. 14.0 % for the younger group, *p* < 0.001),were also more frequent in the elderly group. Mean Disease Activity Score (DAS) was calculated based on three variables, including 28- swollen and tender joints count and erythrocyte sedimentation rate (ESR). Mean DAS at start of biologic DMARDs did not differ between the two groups (Table 1).

**Table 1:** Comparison of demographic and clinical characteristics in elderly and younger patients treated with TNF inhibitors

	Total (*n* = 429)	Elderly patients (*n* = 107)	Younger patients (*n* = 322)	*p*-value
**Age, year**	**49.5 ± 13.6**	**66.1 ± 5.4**	**44.0 ± 10.6**	**<0.01**
**Sex, male**	**60 (14.0)**	**24 (22.4)**	**36 (11.2)**	**0.01**
**Disease duration, years**	**8.6 ± 6.8**	**10.0 ± 7.9**	**8.2 ± 6.4**	**0.03**
First use of TNF inhibitor	329 (76.7)	83 (77.6)	246 (76.4)	0.91
**Number of previous non-biologic DMARDs used**	**4.1 ± 1.5**	**4.4 ± 1.7**	**3.9 ± 1.5**	**0.01**
Concomitant MTX use	311 (72.5)	79 (73.8)	232 (72.1)	0.75
Concomitant MTX dosage, mg/week	14.0 ± 3.8	13.6 ± 3.5	14.2 ± 3.6	0.20
Concomitant glucocorticoid use	331 (77.2)	83 (77.6)	248 (77.0)	1.00
Concomitant glucocorticoid dosage, mg/day	6.0 ± 4.1	6.5 ± 5.8	5.8 ± 3.4	0.34
RF positivity	327 (76.2)	84 (78.5)	243 (75.5)	0.59
DAS28-ESR*	6.0 ± 0.9	6.0 ± 0.8	6.0 ± 0.9	0.46
Comorbid conditions				
** Cardiovascular disease**	**12 (2.8)**	**8 (7.5)**	**4 (1.2)**	**<0.01**
** Pulmonary disease** ^**a**^	**23 (5.4)**	**17 (15.9)**	**6 (1.9)**	**<0.01**
Previous history of pulmonary tuberculosis	41 (9.6)	14 (13.1)	27 (8.4)	0.21
Gastrointestinal disease	138 (32.2)	41 (38.3)	97 (30.1)	0.15
Hepatobiliary disease^b^	46 (10.7)	13 (12.2)	33 (10.3)	0.71
** Diabetes mellitus**	**41 (9.6)**	**20 (18.7)**	**21 (6.5)**	**<0.01**
Malignancy	18 (4.2)	7 (6.5)	11 (3.4)	0.17
** Hypertension**	**95 (22.1)**	**50 (46.7)**	**45 (14.0)**	**<0.01**
Thyroid disease	26 (6.1)	7 (6.5)	19 (5.9)	0.99
Renal disease^c^	10 (2.3)	1 (0.9)	9 (2.8)	0.46
Biologic agents				
Etanercept	264 (61.5)	72 (67.3)	192 (59.6)	0.34
Adalimumab	113 (26.3)	25 (23.4)	88 (27.3)	
Infliximab	52 (12.1)	10 (9.4)	42 (13.0)	

^a^Pulmonary disease: interstitial lung disease, asthma, or chronic obstructive pulmonary disease, ^b^Hepatobiliary disease: hepatitis B, hepatitis C, fatty liver, liver cirrhosis; ^c^Renal disease: Modification of Diet in Renal Disease (MDRD) GFR < 15

*TNF* tumor necrosis factor, *RA* rheumatoid arthritis, *MTX* methotrexate, *DMARD* disease modifying anti-rheumatic drug, *DAS28* disease activity score with 28 joint assessment, *ESR* erythrocyte sedimentation rate

*DAS28ESR(3) is the disease activity score calculated from three variables including tender joint count, swollen joint count, and ESR

Bold means statistical significant at the *p* < 0.05

### Drug retention rate of TNF inhibitor in elderly patients

**Fig. 1:**
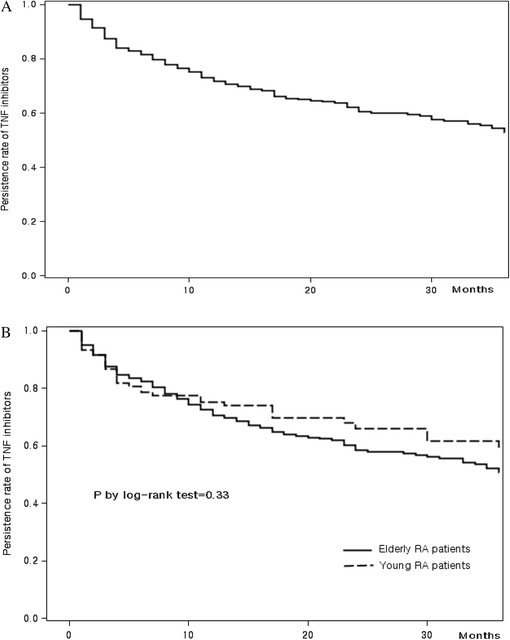
Persistence rate of TNF inhibitors. **a** Persistence rate of TNF inhibitors in total patients. **b** Comparison of TNF inhibitor persistence rate between elderly patients and younger patients

In the analysis of TNF inhibitor retention rates, there was no significant difference over three years between the elderly and young groups (59.0 % in elderly group, 50.8 % in younger group, *p* = 0.33 by log-rank test) (Fig. 1). The median (interquartile range [IQR]) treatment period for each group was 2.0 (95 % CI 0.3–2.6) years for the elderly group and 1.9 (95 % CI 0.5–2.8) years for the younger group. The numbers of patients who discontinued TNF inhibitors for any reason during the observation period were 35 (32.7 %) for the elderly group and 132 (41.0 %) for the younger group (*p* = 0.16 by chi-square test). There was a difference in the frequency of discontinuation by reason: development of AE (34.3 %) was the most common reason in the elderly group, while ineffectiveness (41.7 %) was the most common reason in the younger group, but this difference was not statistically significant (Table 2).

**Table 2:** Reasons for discontinuation of TNF inhibitors, N (%)

	Total (*n* = 429)	Elderly patients (*n* = 107)	Younger patients (*n* = 322)	*p* value
Number of discontinuations	167 (38.9)	35 (32.7)	132 (41.0)	0.16
Reason for discontinuation				0.43
**Adverse effect**	**44 (26.4)**	**12 (34.3)**	**32 (24.2)**	
**Ineffectiveness**	**64 (38.3)**	**9 (25.7)**	**55 (41.7)**	
Patient need	37 (22.2)	9 (25.7)	28 (21.2)	
Good effectiveness	7 (4.2)	2 (5.7)	5 (3.8)	
Economic problem	3 (1.8)	1 (2.9)	2 (1.5)	
Operation or hospitalization	4 (2.4)	1 (2.9)	3 (2.3)	
Other^a^	3 (1.8)	1 (2.9)	2 (1.5)	
Unknown	5 (3.0)	-	5 (3.8)	

^a^Other reasons include mobility impaired, preparation for pregnancy, and dental treatment

Bold means statistical significant at the *p* < 0.05

Risk factors for TNF inhibitor discontinuation in the elderly group were different from those in the younger group. A predictor of TNF inhibitor discontinuation in the elderly group was increasing age (hazard ratio [HR] 1.09, CI 1.02 - 1.16), while the presence of a comorbidity (HR 0.37, CI 0.15-0.91) was a protective factor. For the younger group, first use of a TNF inhibitor (vs. switcher, HR 1.75, CI 1.06-2.94) was a predictor for discontinuation, while longer disease duration (HR 0.97, CI 0.94 – 0.997) had a protective effect against discontinuation of TNF inhibitors (Table 3).

**Table 3:** Risk factors for TNF inhibitor discontinuation in RA patients

	Total RA		Elderly patients		Young RA	
	Adjusted HR (95 % CI)	*p*	Adjusted HR (95 % CI)	*p*	Adjusted HR (95 % CI)	*p*
Age at start of biologics	1.00 (0.99 – 1.02)	0.66	**1.09 (1.02 – 1.16)**	<0.01	1.00 (0.99 – 1.02)	0.64
Sex, female	1.06 (0.65 – 1.74)	0.81	1.81 (0.64 – 5.12)	0.27	0.84 (0.47 – 1.49)	0.55
Switcher vs. first user	**0.55 (0.35 – 0.86)**	<0.01	0.43 (0.16 – 1.22)	0.11	**0.57 (0.34 – 0.94)**	0.03
Disease duration, per year	**0.97 (0.95 – 0.997)**	0.03	0.96 (0.91 – 1.00)	0.05	**0.97 (0.94 – 0.997)**	0.03
Biologics						
Etanercept	1.00		1.00		1.00	
Infliximab	1.44 (0.92 – 2.27)	0.11	0.43 (0.10 – 1.97)	0.28	**1.81 (1.11 – 2.95)**	0.02
Adalimumab	0.88 (0.59 – 1.32)	0.54	0.72 (0.30 – 1.74)	0.46	0.86 (0.54 – 1.37)	0.53
Concomitant use of methotrexate	0.72 (0.51 – 1.01)	0.06	**0.44 (0.21 – 0.91)**	0.03	0.91 (0.62 – 1.35)	0.64
Concomitant glucocorticoid dosage						
<5 mg/day	1.00		1.00		1.00	
≥5 mg/day	1.05 (0.76 – 1.46)	0.75	1.18 (0.53 – 2.62)	0.68	0.97 (0.67 – 1.41)	0.89
Comorbidity^a^	0.91 (0.64 – 1.29)	0.59	**0.37 (0.15 – 0.91)**	0.03	1.04 (0.71 – 1.52)	0.83

*TNF* tumor necrosis factor, *HR* hazard ratio, *CI* confidence interval, *RA* rheumatoid arthritis

^a^Comorbidity: the presence of a comorbid condition

Bold means statistical significant at the *p* < 0.05

### Safety of TNF inhibitors in elderly RA patients

The types and occurrence of SAEs were evaluated. Among 0107 elderly RA patients with 212 PYs, 13 SAEs were reported during the observation period, while 32 SAEs were found in 322 younger patients with 626 PYs. Infection, injury and malignancy were common events in elderly RA patients (five cases, two cases and two cases, respectively), while infection and malignancy were the most common event in younger patients (15 and six cases). The incidence rate of SAEs was slightly higher in the elderly group (6.13/100 PYs) than in the younger group (5.11/100 PYs). The IRs of injury (0.94/100 PYs) and respiratory disorder (0.47/100 PYs) for the elderly group were higher than those of the younger group (0.48/100 PYs and 0.16/100 PYs, respectively). The crude IRR comparing elderly RA patients with young RA patients for all SAEs was 1.20 (95 % CI, 0.63-2.29). The IRRs for injury and respiratory disorder were 1.97 (95 % CI, 0.33-11.78) and 2.95 (95 % CI, 0.18-47.21), respectively, (Table 4).

**Table 4:** Incidence rates (95 % CI) of severe adverse events among RA patients

System organ class allocation	Total (838 PYs)	Elderly patients (212 PYs)	Younger patients (626 PYs)	IRR (elderly patient/younger patients)
Total	**5.37 (3.92 – 7.19)**	**6.13 (3.27 – 10.49)**	**5.11 (3.50 – 7.22)**	**1.20 (0.63–2.29)**
General disorders	0.12 (0.00 – 0.66)	0.47 (0.01 – 2.63)	–	
Hypersensitivity	0.24 (0.03 – 0.86)	–	0.32 (0.04 – 1.15)	
Hepatobiliary disorders	0.48 (0.13 – 1.22)	0.47 (0.01 – 2.63)	0.48 (0.10 – 1.40)	0.98 (0.10–9.46)
Infections	2.39 (1.46 – 3.69)	2.36 (0.77 – 5.50)	2.40 (1.34 – 3.95)	0.98 (0.36–2.71)
Injury	**0.60 (0.19 – 1.39)**	**0.94 (0.11 – 3.41)**	**0.48 (0.10 – 1.40)**	**1.97 (0.33–11.78)**
Musculoskeletal and connective tissue disorders	0.24 (0.03 – 0.86)	–	0.32 (0.04 – 1.15)	
Malignant	0.95 (0.41 – 1.88)	0.94 (0.11 – 3.41)	0.96 (0.35 – 2.09)	0.98 (0.20–4.88)
Nervous system disorders	0.12 (0.00 – 0.66)	0.47 (0.01 – 2.36)	–	
Respiratory, thoracic, and mediastinal disorders	**0.24 (0.03 – 0.86)**	**0.47 (0.01 – 2.36)**	**0.16 (0.00 – 0.89)**	**2.95 (0.18–47.21)**

*CI* confidence interval, *RA* rheumatoid arthritis, *PYs* patient–years, *IRR* incidence rate ratio. Values are Incidence per 100 PYs

Bold means statistical significant at the *p* < 0.05

## Discussion

This study demonstrates that the retention rate of TNF inhibitors in elderly RA patients is comparable to that of younger patients in clinical practice. However, major causes of drug discontinuation differed, with development of AEs in elderly patients and ineffectiveness in younger patients. The predictors of TNF inhibitor discontinuation also differed. Predictors for discontinuation were glucocorticoid use and older age in elderly RA patients while first use of TNF inhibitor and short disease duration were predictors in younger patients. The incidence of overall SAEs in elderly RA patients was higher than that in younger patients, with a IRR of 1.22.

Retention rates of 71.7 % after 1 year, 60.4 % after 2 years, and 52.7 % after 3 years were observed in RA patients. Elderly patients had a similar TNF inhibitor retention rate at 3 years compared with younger patients, with retention rates of 75.2 % for one year, 66.1 % for two years, and 59.0 % for three years, respectively. Previous observational studies reported the good effectiveness of TNF inhibitors in elderly RA patients using outcomes of disease activity changes or functional disability [6, 15–18]. A Dutch registry reported that elderly patients with RA exhibit reduced response to treatment with TNF inhibitors [15]. We cannot directly compare our results with those of previous reports because we did not estimate response rates based on ACR 20 or DAS28. However, our results showed similar retention rates for TNF inhibitors at three years in the elderly group and younger group despite a higher rate of SAEs in elderly patients.

The most common cause of discontinuation differed for elderly and younger groups. AE was the most common cause of discontinuation in the elderly patients, while ineffectiveness was the most common cause in the younger group. This tendency was also demonstrated in an Italian registry in which discontinuation due to AEs (21.8 %) was more frequent than discontinuation due to ineffectiveness (17.4 %) in elderly patients [16].

The presence of comorbidity showed a protective effect for discontinuation of TNF inhibitors in the elderly group. This result was somewhat different from that of a previous study reporting that increased age was associated with a greater number of comorbidities and suggesting that comorbidities were associated with poorer response outcomes for disease activity or functional disability [19]. Since comorbidity affects not only treatment outcomes but also treatment decisions [20], it might be associated with persistence of TNF inhibitors in a different way. In some previous studies, greater comorbidity was associated with greater discontinuation rate of TNF inhibitors [21–23], whereas other studies noted a positive influence of comorbidities on the drug persistence [24, 25]. RA patients with biologic DMARDs have been shown to have high levels of baseline comorbidity [26]. However, TNF inhibitors have restricted applications in some comorbid conditions, such as congestive heart failure, infection, and malignancy. Therefore, in accordance with our study, comorbid conditions in RA patients that are treated with TNF inhibitors in clinical practice may not lead to drug discontinuation and contribute to maintain TNF inhibitor treatment because these injectable agents reduce the number and/or dose of medications, such as glucocorticoid and immunosuppressive agents. In the younger group, first use and short disease duration were related to discontinuation of TNF inhibitors. This may indicate that intensive drug switching is more common in younger patients than elderly patients, since they are more socially active.

The IR of overall SAE in elderly patients was numerically higher than in the young patients (IRR 1.20, 95 % CI 0.63-2.29). Among various SAEs, the incidence of injury and respiratory disorder in elderly patients was higher than those in young patients. Previous observational studies have reported that elderly patients were more susceptible to infection [16, 27–30]. These results may be from more risk factors for infection in elderly RA patients, including higher prevalence of comorbidity, elevated markers of disease activity, and history of previous infection [31]. The malignancy risk in elderly patients receiving TNF inhibitors is controversial. In a Swiss registry, malignancy risk was higher in elderly users of TNF inhibitors [18], but other observational studies showed that there was no increase in the risk of malignancy in elderly patients treated with TNF inhibitors [31, 32]. In subgroup analysis for elderly patients more than 65 years old of our results, infection and malignancy risks were higher in the elderly group than in the younger group, but the difference was not statistically significant (Additional file 1: Table S1). Although this encouraging result is from a small number of elderly patients and a relatively short observational period, we could insist that certain selection criteria for TNF inhibitors in elderly RA patients are reasonable in real-world clinical practice.

Our study includes certain limitations. First, the current study includes a small number of elderly patients. Further observational study using multiple data sources is needed to evaluate long-term effectiveness and safety for elderly RA patients. Second, we did not consider elderly onset patients because patients were classified based on the age when they started TNF inhibitors. Effectiveness and safety in elderly onset patients may be different because their disease characteristics are different. Elderly onset RA is more often associated with joint erosions, higher disease activity, and higher HAQ scores at baseline [33]. However, initial aggressive treatment or multiple combinations can be difficult to apply in elderly onset RA patients. Therefore, the effectiveness and safety of TNF inhibitors should be studied. Thirdly, we could not evaluate retention rate and safety for each TNF inhibitor because of our small population. Since so many TNF inhibitors, including biosimilars and biologic DMARDs with other mechanisms, are available worldwide, further effort should invested in identifying a strategy for appropriate selection of biologic DMARDs for elderly RA patients.

## Conclusions

The retention rate of TNF inhibitors in the elderly RA group was comparable to that in the younger group. The major cause of discontinuation in elderly patients was AE, while the major cause of discontinuation in younger patients was ineffectiveness. The IRs of injury and respiratory disorder in elderly patients were higher than those in younger patients, but the differences were not statistically significant.

## Abbreviations

RA, Rheumatoid arthritis, TNF: tumor necrosis factor, AEs: adverse events, DMARDs: disease-modifying anti-rheumatic drugs, SAEs: Serious adverse events, SOC: System Organ Class, IR: incidence rate, IRR: incidence rate ratio, PSL: prednisolone, IQR: interquartile range, OR: odds ratio

## Additional file

Additional file 1: Table S1. Incidence rates (95 % CI) of severe adverse events among RA patients; subgroup analysis for more elderly patients ≥ 65 years old. (DOCX 15 kb)
