# Supplementary material for: Drug retention and safety of TNF inhibitors in elderly patients with rheumatoid arthritis
Source: BMC Musculoskelet Disord. 2016 Aug 9;17:333. doi: 10.1186/s12891-016-1185-6 (PMC4977640; doi:10.1186/s12891-016-1185-6)
Supplement: Additional file 1: Table S1. — Incidence rates (95 % CI) of severe adverse events among RA patients; subgroup analysis for more elderly patients ≥ 65 years old. (DOCX 15 kb) [file 12891_2016_1185_MOESM1_ESM.docx]

Additional file 1: Table S1. Incidence rates (95% CI) of severe adverse events among RA patients; subgroup analysis for more elderly patients≥65 years old

| System Organ Class Allocation | Total  (838 PYs) | Elderly patients  (110 PYs) | Younger patients  (728 PYs) | IRR  (elderly patient/younger patients) |
| --- | --- | --- | --- | --- |
| Total | **5.37 (3.94 - 7.12)** | **6.36 (2.60 – 12.67)** | **5.22 (3.72 - 7.09)** | **1.22 (1.38-2.99)** |
| General disorders | 0.12 (0.01 – 0.66) | 0.91 (0.02 - 4.96) | - |  |
| Hypersensitivity | 0.24 (0.03 - 0.86) | - | 0.27 (0.03 - 0.99) |  |
| Hepatobiliary disorders | 0.48 (0.13 - 1.22) | - | 0.55 (0.15 - 1.40) |  |
| Infections | **2.39 (1.46 - 3.66)** | **2.73 (0.57 – 7.76)** | **2.34 (1.37 - 3.71)** | **1.17 (0.55-3.78)** |
| Injury | 0.60 (0.19 - 1.39) | - | 0.69 (0.22 - 1.60) |  |
| Musculoskeletal and connective tissue disorders | 0.24 (0.03 - 0.86) | - | 0.27 (0.03 - 0.99) |  |
| Malignant | **0.95 (0.41 – 1.87)** | **1.82 (0.22 – 6.41)** | **0.82 (0.30 – 1.79)** | **2.21 (0.92-3.05)** |
| Nervous system disorders | 0.12 (0.01 - 0.66) | 0.91 (0.02 – 4.96) | - |  |
| Respiratory, thoracic, and mediastinal disorders | **0.24 (0.03 - 0.86)** | **-** | **0.27 (0.03 - 0.99)** |  |

* CI, confidence interval; RA, rheumatoid arthritis; PYs, patient-years; IRR, incidence rate ratio. Values are Incidence per 100 PYs.
